# Relation between stressful life events and psychosis

**DOI:** 10.1192/j.eurpsy.2023.2212

**Published:** 2023-07-19

**Authors:** A. Osca Oliver, R. Pérez Iglesias, M. V. López Rodrigo, V. Ros Fons, Y. D´Hiver, G. Sánchez, M. Pérez Fominaya

**Affiliations:** 1 Hospital Nuestra Señora del Prado, Talavera de la Reina; 2 Hospital Universitario Valdecillas, Santander, Spain

## Abstract

**Introduction:**

Numerous studies establish clear connections between traumatic childhood experiences and the risk of developing psychosis. According to the study carried out by Filippo Varese, childhood traumas, understanding by; physical, psychological or sexual abuse, abandonment, death of the parent and “bullying”, increase up to three times the risk of suffering from psychosis.

**Objectives:**

- Determine the prevalence of traumatic events in the sample studied.
- Determine which traumatic event has a greater relationship with the risk of presenting a psychotic episode.
- To determine whether traumatic events may be more strongly associated or not with gender differences, age at onset and family story.

**Methods:**

A descriptive study is carried out in which the traumatic events are evaluated (through the CAVE questionnaire) of 98 patients who have been treated for a psychotic episode in the last two years in the Early Intervention Unit for Psychosis (ITPCan).

The stressful life history questionnaire (CAVE) consists of 52 questions divided into blocks: school stage, work, partner, family, health and other stressful events. All of them focused on those stressful events prior to the onset of psychotic symptoms. Stressful events have been considered to be those events in which the patients studied have scored 10 (maximum score) on the anxiety scale.

Inclusion criteria:

- Older than 18 years-old
- Having presented a first psychotic episode.

Exclusion criteria:

- Intellectual disabilities

**Results:**

For now, the data studied in this sample are similar to those described in most of the studies reviewed: more than half of the patients present at least one traumatic event before the onset of psychotic symptoms and a third of these have had any traumatic experience before the age of 18.

The percentage of the presence of at least one traumatic event within the categories of the CAVE questionnaire would be:

14.2% in the school stage

26.7% in the workplace

26% in relationships

24.3% in family relationships

12.1% in events related to the patient’s own health problems

**Conclusions:**

We continue to increase the sample to have a more significant result.

**Disclosure of Interest:**

None Declared

